# Efficacy of a mobile-based self-directed parent management training for parents of children with attention-deficit/hyperactivity disorder with or without oppositional defiant disorder– a randomized controlled trial

**DOI:** 10.1007/s00787-025-02799-2

**Published:** 2025-06-27

**Authors:** M. Döpfner, A. Görtz-Dorten, A. Häge, F. Handermann, L. Sulprizio, M. Hellmich, D. Vogel, M. Ruhmann, H. Althen, M. Karus, T. Banaschewski

**Affiliations:** 1 Centre of Child and Adolescent Cognitive Behavior Therapy (CEKIP), Faculty of Medicine and University Hospital Cologne, University of Cologne, Cologne, Germany; 2 Department of Child and Adolescent Psychiatry, Psychosomatics and Psychotherapy, Faculty of Medicine and University Hospital Cologne, University of Cologne, Cologne, Germany; 3 Department of Child and Adolescent Psychiatry and Psychotherapy, Central Institute of Mental Health, Medical Faculty Mannheim, Heidelberg University, Mannheim, Germany; 4 Institute of Medical Statistics and Computational Biology, Faculty of Medicine and University Hospital Cologne, University of Cologne, Cologne, Germany; 5 Department of Medical Statistics, University Medical Center Göttingen, Göttingen, Germany; 6 Medigital GmbH, Iserlohn, Germany; 7 MEDICE Arzneimittel Pütter GmbH & Co. KG, Iserlohn, Germany; 8 German Center for Mental Health (DZPG), partner site Mannheim- Heidelberg-Ulm, Mannheim, Germany

**Keywords:** Attention-deficit/hyperactivity disorder, Oppositional defiant disorder, Parent management training, Digital health application, Digital therapeutic, Randomized controlled trial

## Abstract

Preliminary empirical evidence supports the efficacy of digital parent management training (d-PMT) in the treatment of externalizing behavior problems in children. This study investigated the efficacy of a mobile-based self-directed d-PMT for parents of children aged 4–11 years with attention-deficit/hyperactivity disorder (ADHD) with or without oppositional defiant disorder (ODD), and without pharmacological and/or behavioral therapy. Participants were randomized 1:1 to d-PMT (hiToco^®^) plus treatment as usual (TAU) or TAU only for 16 weeks. The primary outcome was a reduction of parent-rated externalizing problem behavior including symptoms of ADHD and ODD. Secondary outcomes included parent-rated ADHD symptoms only, ODD symptoms only, and functional impairment of the child as well as parenting behavior and family strain. Sixty-five participants were randomized to the dPMT + TAU group (*n* = 34) or the TAU group (*n* = 31). All children had a clinical diagnosis of ADHD; clinically diagnosed ODD was reported in 9 children (26.5%) in the d-PMT + TAU group and 13 children (42%) in the TAU group. The mean ± SD age of the children was 8.4 ± 1.7 years (69.2% boys). On average, participants used the program for 8 h in total. Regarding the primary outcome, significant treatment effects favoring the d-PMT + TAU group compared to the TAU group were observed at week 12 (W12) and W16 (Cohen’s d at W12: 0.74; at W16: 0.48). Post-hoc analyses showed that 50% of the children in the d-PMT + TAU group compared to 30% in the TAU group were reliably recovered or improved. Significant treatment effects in favor of the d-PMT + TAU group were also observed for all secondary variables. Our findings suggest that a mobile-based self-directed d-PMT for parents may play an important role in the multimodal treatment of children with ADHD in future clinical routine practice.

**Supplementary Information:**

The online version contains supplementary material available at 10.1007/s00787-025-02799-2.

## Trial registration

German Registry of Clinical Studies Identifier DRKS00030086 (registered on 12 September 2022).

**Supplementary Information:**

The online version contains supplementary material available at 10.1007/s00787-025-02799-2.

## Introduction

Behavioral parent management training (PMT) has proven effective in the treatment of externalizing behavior disorders such as attention-deficit/hyperactivity disorder (ADHD) and oppositional defiant disorder (ODD) in children [1, 2]. In line with these observations, current German clinical practice guidelines recommend this intervention as first-line therapy for both preschoolers and school-aged children with mild to moderate ADHD symptom burden [3]. Self-directed parenting interventions, such as workbook-based or mobile-based PMT, may overcome frequently reported barriers to accessing face-to-face treatment, including limited local service availability, fear of stigmatization, or limited time and financial resources. Moreover, the frequently reported lack of resources for face-to-face PMT [4] may be alleviated, thereby decreasing waiting times for treatment and improving overall coverage with PMT for the treatment of ADHD and other externalizing disorders. Individual psychotherapy, the dominant model of treatment delivery, is not likely to be able to meet treatment needs [5].

Recent meta-analyses have demonstrated that digital PMT (d-PMT), either alone or in combination with therapist support, including self-directed interventions, has small to moderate effects on child behavior problems and child emotional problems, mostly small effects on negative discipline strategies, parenting efficacy, parenting confidence, parent distress, parenting satisfaction, and aspects of parental mental health, and large effects on positive parenting behaviors and the quality of parent-child interactions [6–9]. Moreover, d-PMT was non-inferior to both self-directed PMT based on written materials [6, 10] and face-to-face PMT [11–13]. Some studies found differences in self-directed d-PMTs with or without additional therapist support, while others found no differences (e.g [14, 15])., In most studies, clinical diagnoses of ADHD and/or ODD were not required for inclusion. Therefore, most studies are either indicated prevention studies or clinical studies conducted in referred samples of children with elevated ratings of externalizing behavior problems but not necessarily a formal diagnosis. Most programs were delivered via a website [6], with only one delivered through a mobile app [16]. Few studies assessed the effects of the intervention on impairment, completion rates or intensity of program use. Moreover, most programs do not tailor the specific interventions to individual family or parent needs, and specific interventions supporting the transfer of trained skills into the daily interactions with the child at home are rarely implemented (e.g., therapeutic homework assignments, monitoring of implementation of interventions, support to overcome implementation barriers experienced in therapeutic homework assignment, positive feedback for the parent).

The objective of this pilot randomized controlled trial was to assess the usage and efficacy of a newly developed d-PMT in children referred for treatment of ADHD and/or ODD problems and with a clinical diagnosis of ADHD and/or ODD. We hypothesized that the d-PMT in addition to treatment as usual (TAU) would be more effective than TAU alone in reducing ADHD and ODD symptoms over a 16-week period. The d-PMT is personalized and tailored to the individual needs of the parents and the specific problem situations in the family. To enhance parental engagement and implementation of interventions in the real world, therapeutic homework assignments, monitoring of the transfer of the interventions to the family, and tailored feedback regarding successful or unsuccessful transfer were also implemented. Efficacy was assessed across several outcome measures, including ADHD and ODD symptoms, impairment, parenting behavior and family strain. Additionally, app usage was monitored. The current study was designed as a pilot study to gain initial insights into the efficacy of the new application in a smaller patient sample. The results of this study will inform the design of a subsequent pivotal study.

## Methods

### Study design

This multicenter, randomized, controlled, parallel-group pilot study was conducted in routine clinical care settings across 10 German local trial sites and one central trial site for telemedical participation throughout Germany. The study compared the efficacy and safety of a newly developed self-directed d-PMT in addition to TAU with TAU alone. The study was carried out in accordance with good clinical practice (DIN EN ISO 14155:2021-05) and the Declaration of Helsinki in its latest version. The study was approved by the Ethics Committee of the Medical Faculty of the University Heidelberg, Germany as leading ethics committee on July 7, 2022. The study was prospectively registered in the German Registry of Clinical Studies (DRKS00030086). The randomized controlled trial reported in this paper was part of a larger study setting that also included two observational studies. These studies examined the effects of the application in two other cohorts: children with a suspected diagnosis of ADHD/ODD and children with ADHD receiving pharmacotherapy but experiencing residual symptoms requiring treatment. The publication of the results of these studies is in preparation.

### Study conduct

Changes to the clinical investigational plan during the study are summarized in Online Resource 1. Recruitment was carried out during routine clinical care of the child either by the local investigator (treating physician) or by a central telemedical investigator after referral by a treating physician who provided a signed report with information about the clinical diagnosis of the child, previous/current treatments and comorbid conditions. Families received written and oral information about the study from an investigator and were free to decide on study participation. All parents/legal guardians had to give written informed consent. The children had to give at least oral assent and were additionally offered the opportunity to add their names on the informed consent form. Upon giving informed consent, families were screened for eligibility, and, if eligible, were enrolled into the study. Demographic, socioeconomic and disorder-specific baseline parameters were documented, and participants received a link to a baseline (T0) survey via email. This survey contained all rating scales used to assess the outcomes listed below. Upon completion, the investigator randomized the participants using a build-in randomization tool of the electronic data capture system. The investigator had no influence on the result of the randomization. Randomization with a 1:1 allocation ratio was carried out separately at each trial site. Allocation sequences were stratified by sex of the child and were based on permuted blocks of varying (4 or 6) length. Participants were informed about their group allocation by the investigator via email. As part of this email, participants randomized to the d-PMT + TAU group received an activation code for the d-PMT. Following activation, access to the app was valid for a total of 180 days. After 8 weeks (T1/W8), 12 weeks (T2/W12) and 16 weeks (T3/W16), all participants were again invited to complete an online survey via email for outcome ratings. At the end of each of these surveys, participants were asked to report any adverse events (AEs) to the investigator. Within three weeks after W16, a final visit with the investigator took place to capture potential further AEs as well as device deficiencies and to document therapeutic measures taken throughout the study. During this visit, children in the d-PMT + TAU group were asked whether they had noticed any changes in their parents’ behavior during the study. The baseline visit and the final visit could be conducted on site or via a telemedicine-based online visit (end-to-end encrypted). Participants were compensated for their participation in the study, and those in the TAU group were given access to the d-PMT after completing the study.

### Participants/patients

Eligible children (patients) were aged between 4;0 and 11;11 years and had a definitive clinical diagnosis of ADHD (International Statistical Classification of Diseases and Related Health Problems, 10th revision [ICD-10]: F90.x, F98.80) and/or ODD (ICD-10: F91.3) documented by the child’s treating physician (i.e., the clinician was certain that the clinical criteria for the diagnosis were met). Furthermore, children had elevated parent-rated symptoms of ADHD and/or ODD (mean score > 1.0 = 78th -89th percentile in age specific representative samples) based on the German *Fremdbeurteilungsbogen* (FBB)-ADHS (Symptom Check List (SLC)-ADHD; parts A and B) and/or FBB-SSV (SCL-ODD; part A) at screening [17, 18]. Parents (participants) were required to have sufficient reading and German language skills, as well as a smartphone or tablet PC with internet connection. Key exclusion criteria included (1) current or planned ADHD medication for the child within the next four months, (2) intensive behavioral therapy for the child or regular parent training both with at least two contacts per month within the last 12 months, currently ongoing or planned within the next four months, (3) another severe mental disorder of the child (e.g., intellectual disability, autism, psychosis), (4) a known severe mental disorder of the participant (e.g., severe substance use disorder, severe emotionally unstable personality disorder, psychosis) and (5) planned (partial) inpatient therapy for the child due to a mental disorder.

### Investigational device

The d-PMT (brand name hiToco^®^) is a CE-marked class I medical device software according to regulation (EU) 2017/745 on medical devices (MDR). This mobile application (for smartphone and tablet) is based on the established German therapy program for children with hyperkinetic and oppositional problem behavior (THOP; *Therapieprogramm für Kinder mit hyperkinetischem und oppositionellem Problemverhalten* [19]) and self-help workbooks for parents that were derived from THOP [20, 21]. The d-PMT is a comprehensive, integrated and personalized program consisting of five modules: (1) psychoeducation on characteristics, causes, and further course of ADHD/ODD, (2) psychoeducation on assessment and interventions, (3) coping with parental challenges (e.g., reducing own stress, improving own self-control, improving own organizational skills, handling partner problems), (4) strengthening family resources and the parent-child relationship, (5) solving child behavior problems at home (parents select one out of four typical problem situations and are guided to analyze the problem and to develop a specific intervention). Transfer tasks aim to help parents implement the interventions developed in modules 3 to 5 at home. Depending on the feedback of the parents regarding implementation success, the parents were positively reinforced or received further support to overcome implementation barriers. In general, there were no spatial or temporal requirements for the use of the app. However, parents were advised to use the app regularly (e.g., two to three times per week).

The total usage time of the app is flexible since the usage is personalized according to the individual needs of the parents. In case of technical issues with the app or problems with the proposed interventions that could not be resolved by the app, parents had the option to seek online support from the manufacturer. In the latter case, feedback was limited to the content of the app and did not include psychotherapeutic or other therapeutic advice. All investigators and treating physicians were given access to the d-PMT to familiarize themselves with the program, but they were not required for the intended use of this self-directed d-PMT during the study. A detailed description of the d-PMT is given in Online Resource 2.

### Treatment as usual

Following guideline recommendations, TAU was understood as a patient-specific therapy that was developed together with the family by the treating physician of the child, taking into account the age of the child, the severity of the symptoms, the family’s wishes, and the current care situation. As stated above, pharmacotherapy or psychological interventions were exclusion criteria. However, if indicated, the treating physician could initiate both pharmacological and non-pharmacological treatments during the study. The investigator documented previous and current treatments at baseline as well as any therapeutic measures taken during the study at the final study visit. Investigators at the central trial site did not provide TAU; this was provided by the treating physician.

### Primary outcome

The primary outcome measure was the mean item score of the 28 items on the parent-rated SCL-ADHD/ODD scale, which includes the 20 items of the SCL-ADHD (parts A and B) and the eight items of SCL-ODD (part A) [18, 22–24]. The combination of both scales was used to measure the externalizing problem behavior of the child. All items were rated on a 4-point Likert scale (0 = not at all, 1 = a little, 2 = to large extent, 3 = extremely). A detailed description of this measure is provided in Online Resource 3.

### Secondary/further outcomes

Parent-rated symptoms of ADHD and oppositional behavior were measured using the 20 items of the SCL-ADHD and the eight items of the SCL-ODD, respectively. Parent-rated functional impairment of the child was assessed using five items of the SCL-ADHD (part F) [17]. Parenting behavior was measured via the Assessment Scale of Positive and Negative Parenting Behavior (FPNE, German: *Fragebogen zum positiven und negativen Erziehungsverhalten*) [25]. Family strain was measured using the Family Strain Index (FSI) [26]. Details of these measures are provided in Online Resource 3.

Participants were advised to report any AEs and device deficiencies throughout the study and were specifically asked by the investigator at the final visit. AEs and device deficiencies were coded according to the Medical Dictionary for Regulatory Activities (MedDRA) version 27.0 and the terminology of the International Medical Device Regulators Forum (IMDRF, Annex A), respectively. Any changes in concomitant ADHD and/or ODD-specific therapies were documented throughout the study. At the final visit, the investigator rated the child’s condition using the German version of the validated Clinical Global Impression-Severity/Improvement scale (CGI-S/I) [27, 28].

App user data from participants who voluntarily consented to tracking were collected to gain insights into user behavior (*N* = 29). If participants opted in for tracking and– for iPhone users– the app-specific settings in the Apple iOS did not block tracking, user data could also be collected using the web-analytics platform matomo (www.matomo.org; *N* = 21), which was hosted by Medigital GmbH. The database was filtered for logins with a duration of at least 10 s to ignore logins that do not reflect any meaningful interaction with the program (e.g., brief reminders). Users who did not interact with the app for more than 30 min were automatically logged off. Based on the available data, the number of logins per user, the single login duration (minutes), the total usage time (minutes) and the period of use (time between registration and last observed login in days) were calculated. The completion rate of the individual training plan (ranging from 0 to 1) was calculated by dividing the number of completed articles by the number of articles that were part of the training plan as defined by the initial questionnaire.

### Sample size

Previous randomized controlled trials evaluating d-PMTs have observed small to moderate effect sizes for ADHD/ODD symptoms [6, 9]. Based on these results, an effect size of Cohen’s d = 0.3 was assumed for this pilot study. Thus, the sample size was calculated as 74 participants based on a two-sample t-test with a one-sided level of significance of 50% and a power of 90%. This pilot study is designed to observe with high probability a true effect in the correct direction. If an effect is observed in the incorrect direction (erroneously), it would be difficult to justify a subsequent (larger) confirmatory trial. Therefore, the sample size rationale serves as a safeguard against such misleading results. Allowing for an assumed pre-post correlation of 0.6, the preliminary sample size amounted to 48 participants. In addition, a dropout rate of 20% was assumed to account for participants with missing data over the course of 16 weeks. Thus, a total of 60 participants were needed for this study (74*(1–0.6^2^)/0.8), i.e., 30 participants per randomized group.

### Statistical methods

Pre-specified statistical analyses were carried out using the statistical software SAS^®^ (version 9.4). Post-hoc analyses were performed using R (version 4.4.1). In general, standard descriptive statistics for continuous and categorial variables were used to describe the study sample at baseline and to summarize AEs and further outcomes (i.e., CGI, therapeutic measures taken, usage data). In a post-hoc analysis, baseline characteristics were screened for significant differences between both randomization groups. For continuous variables (e.g., age), the two-sample t-test was used. For categorial variables (e.g., gender, educational qualification), a chi-square-test for independence was used. Analyses of primary and secondary efficacy variables were based on the intention-to-treat (ITT) population, which included all randomized participants and patients. Analyses of baseline data, secondary variables of safety (AEs, device deficiencies) and other variables were based on the safety population, which included all participants/patients who provided oral/written informed consent and were included in the study.

Inferential statistics were used to model between-group as well as within-group changes in the SCL-ADHD/ODD, SCL-ADHD, SCL-ODD, SCL-ADHD part F, FPNE and FSI scores. For that purpose, a linear mixed model for repeated measures (LMMRM) was used, with the change from baseline at each timepoint (W8, W12, W16) as dependent variable. Fixed effects were baseline value, group, time, and interaction group*time. Least squares (LS) means and the differences thereof were calculated as estimators for the within-group and between-group comparison, respectively, and displayed including the 95% confidence intervals (CI). This model contained data from all participants with at least one follow-up value. For the primary efficacy variable, sensitivity analyses were performed to investigate the robustness of the results under the not missing-at-random assumption by controlled multiple imputation [29]. Effect sizes (Cohen’s d) were calculated post-hoc based on the respective raw value for the change from baseline. For between-group effect sizes, the pooled standard deviation (SD) was used. An effect size of 0.2 was considered small, while effect sizes of 0.5 and 0.8 were considered moderate and large, respectively [30].

To address the clinical relevance of changes in the SCL-ADHD/ODD, post-hoc analyses were performed using the reliable change index (RCI) [31]. This method represents an established approach to address clinical relevance in the ADHD field [32–34]. The RCI indicates whether a change in an outcome variable is large enough to reflect a real change considering the precision of the respective instrument (here, the internal consistency of the SCL-ADHD/ODD). Combining this information with normative comparisons allows for classification of the observed changes. In this analysis, we used an SCL-ADHD/ODD mean item score of 1.0 as a cut-off to distinguish between a non-clinical (< 1.0) and a clinical (≥ 1.0) symptom severity, which corresponds to the 85th percentile in the normative sample [17]. This classification ultimately results in four different groups: (I) reliably recovered (reliable mean item score reduction and mean item score at W12/W16 below 85th percentile), (II) reliably improved (reliable mean item score reduction but mean item score at W12/W16 above 85th percentile), (III) no reliable change (no reliable mean item score reduction), (IV) reliably deteriorated (reliable mean item score increase). The analysis was conducted at W12 and W16 in a population restricted to children with an SCL-ADHD/ODD mean item score ≥ 1.0, as well as in the ITT population. Children with missing values at W12 and/or W16 were classified as “no reliable change”. Children classified as “reliably recovered” or “reliably improved” were considered responders, and the Pearson chi-square test was used to assess differences in the responder proportions between the d-PMT + TAU and TAU groups.

## Results

### Participant flow

**Fig. 1:**
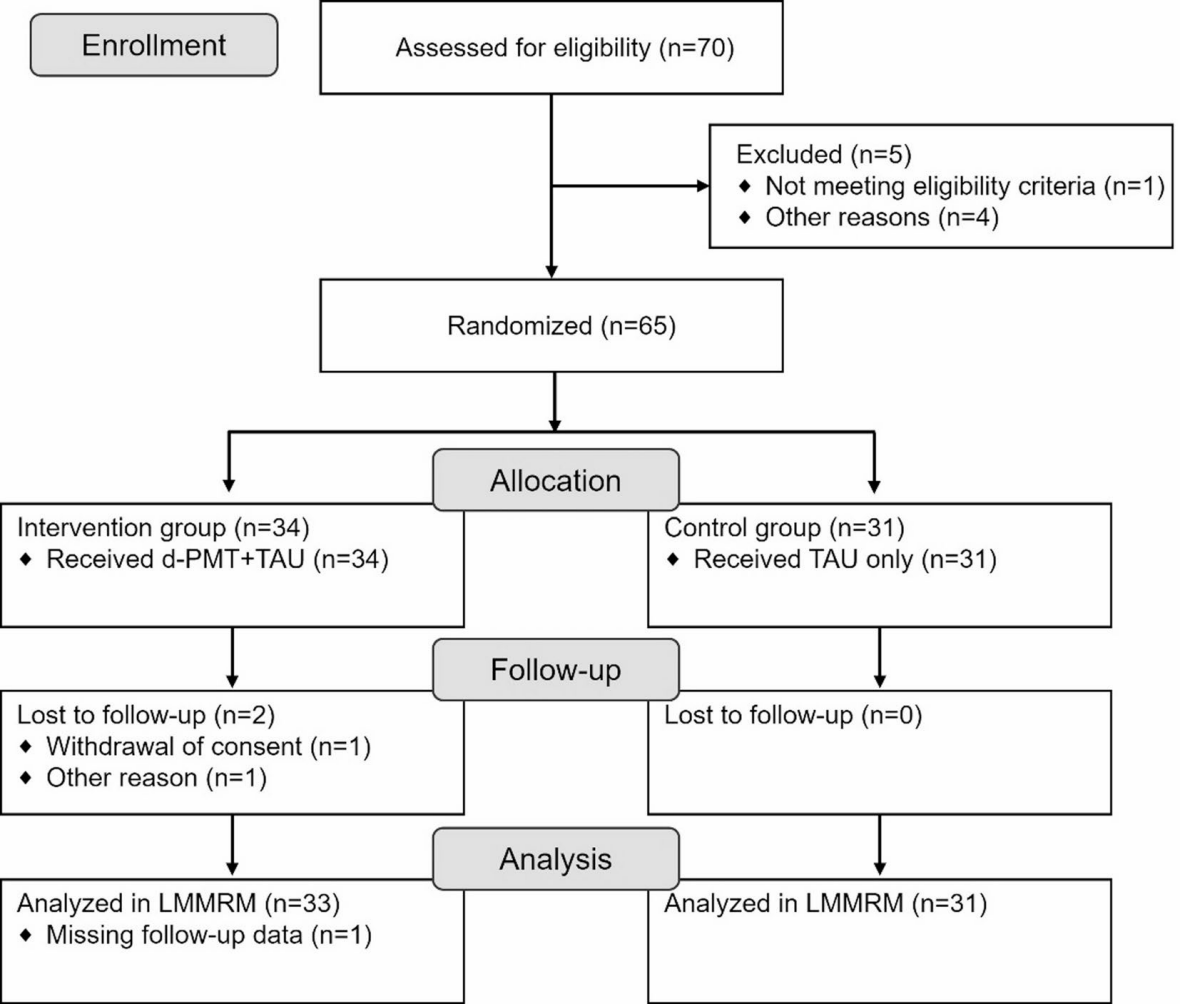
CONSORT flow chart. d-PMT = digital parent management training; LMMRM = linear mixed model of repeated measures; TAU = treatment as usual

Overall, 70 families passed the pre-screening and were screened for eligibility between October 2022 and January 2024, and 65 participating parents and their children were randomized to the d-PMT + TAU group (*n* = 34) and TAU group (*n* = 31). Two participants in the d-PMT + TAU group discontinued the study prematurely (withdrawal of consent; *n* = 1; other reasons; *n* = 1). For statistical analysis using the LMMRM, *n* = 33 and *n* = 31 data sets were available in the d-PMT + TAU group and in the TAU group, respectively (Fig. 1). The last participant completed the study in May 2024. The number of available participants for each assessment phase is shown in Online Resource 4.

### Baseline characteristics

Baseline demographic, sociodemographic and clinical characteristics of the participants and patients are shown in Table 1 and Online Resource 5. Across both study groups, the mean ± SD age of the children was 8.4 ± 1.7 years (69.2% boys). All children had a definitive diagnosis of ADHD. The proportion of children who additionally showed oppositional behavior in a clinical range (“ADHD and ODD”) was slightly lower in the d-PMT + TAU group compared to the TAU group (26.5% vs. 41.9%). At baseline, the SCL-ADHD/ODD mean item score was comparable between both groups (d-PMT + TAU: 1.53 ± 0.42 vs. TAU: 1.64 ± 0.49). The mean ± SD age of the participants was 39.3 ± 4.6 years; most participating parents were women (92.3%). A high proportion of participants (52 to 71%) had a higher education entrance qualification. Occupational therapy was the most common current treatment at baseline.

**Table 1:** Baseline characteristics

	TAU (*N* = 31)	d-PMT + TAU (*N* = 34)	*p*-value^a^
Child			
Age (years)			0.223
Mean ± SD	8.6 ± 1.6	8.1 ± 1.7	
Median (range)	8.5 (5.3–11.9)	8.3 (4.9–11.3)	
Sex, n (%)			1.000
Male	21 (67.7)	24 (70.6)	
Female	10 (32.3)	10 (29.4)	
Diagnosis			0.292^b^
ADHD only, n (%) (ICD-10: F90.0, F90.8, F98.80)	18 (58.1)	25 (73.5)	
- F90.0 (combined presentation)	27 (79.4)	21 (67.7)	
- F90.8 (predominantly hyperactive/impulsive presentation	1 (2.9)	0 (0)	
- F98.80 (predominantly inattentive presentation)	1 (2.9)	1 (3.2)	
ADHD and ODD, n (%) (ICD-10: F90.1 or F90.0 + F91.3)	13 (41.9)	9 (26.5)	
ODD only, n (%) (ICD-10: F91.3)	0 (0)	0 (0)	
Other previous or current psychiatric comorbidities, n (%)	7 (22.6)	7 (20.6)	1.000
Parent ratings at baseline, Mean ± SD			
SCL-ADHD/ODD	1.64 ± 0.49	1.53 ± 0.42	0.336
SCL-ADHD	1.73 ± 0.52	1.59 ± 0.46	0.269
SCL-ODD	1.42 ± 0.73	1.38 ± 0.64	0.796
FPNE pos.	2.09 ± 0.50	1.95 ± 0.40	0.219
FPNE neg.	0.98 ± 0.37	1.01 ± 0.30	0.795
FSI	10.3 ± 4.3	10.0 ± 3.6	0.818
SCL-ADHD part F	1.61 ± 0.62	1.33 ± 0.62	0.077
**Participating parent**			
Age (years)			0.769
Mean ± SD	39.2 ± 4.6	39.5 ± 4.6	
Median (range)	40.0 (28–48)	39.0 (32–49)	
Gender, n (%)			1.000
Male	2 (6.5)	3 (8.8)	
Female	29 (93.5)	31 (91.2)	
Diverse	0 (0)	0 (0)	
Highest educational qualification in the household, n (%)^c^			0.051
No secondary school certificate	0 (0)	1 (2.9)	
Secondary school certificate (Hauptschule)	2 (6.5)	0 (0)	
Secondary school certificate (Realschule)	8 (25.8)	1 (2.9)	
Higher education entrance qualification (Gymnasium)	16 (51.6)	24 (70.6)	
Other	1 (3.2)	3 (8.8)	
No answer	4 (12.9)	5 (14.7)	

ADHD = attention-deficit/hyperactivity disorder; d-PMT = digital parent management training; FPNE neg. = negative-inadequate parenting scale from the Assessment Scale of Positive and Negative Parenting Behavior; FPNE pos. = positive parenting scale from the Assessment Scale of Positive and Negative Parenting Behavior; FSI = Family Strain Index; ICD-10 = International Statistical Classification of Diseases and Related Health Problems, 10th Revision; ODD = oppositional defiant disorder; SCL-ADHD = Symptom Checklist-Attention-Deficit/Hyperactivity Disorder; SCL-ADHD/ODD = Symptom Checklist-Attention-Deficit/Hyperactivity Disorder/Oppositional Defiant Disorder; SCL-ODD = Symptom Checklist-Oppositional Defiant Disorder; SD = standard deviation; TAU = treatment as usual

^a^ The p-values for differences in mean (e.g., age) are obtained by two-sided two-sample t-test. The p-values for differences in proportions (e.g., sex, diagnosis) are based on the chi-square-test for independence of the margins in a contingency table

^b^ The p-value refers to the comparison of ADHD only, ADHD and ODD, and ODD only

^c^ Secondary education based on German school system: Hauptschule (“lower” secondary school, 9 years), Realschule (“middle” secondary school, 10 years), and Gymnasium (“higher” secondary school, 12 or 13 years)

### App usage

An overview of app usage based on the available data sets (*n* = 21) is given in Online Resource 6. On average, participants used the app for 119.4 ± 25.0 days with a mean ± SD number of logins of 35.0 ± 22.1. The average total usage time was 498.6 ± 269.3 min, and the median training plan completion rate was 85%.

### Primary efficacy outcome

**Fig. 2:**
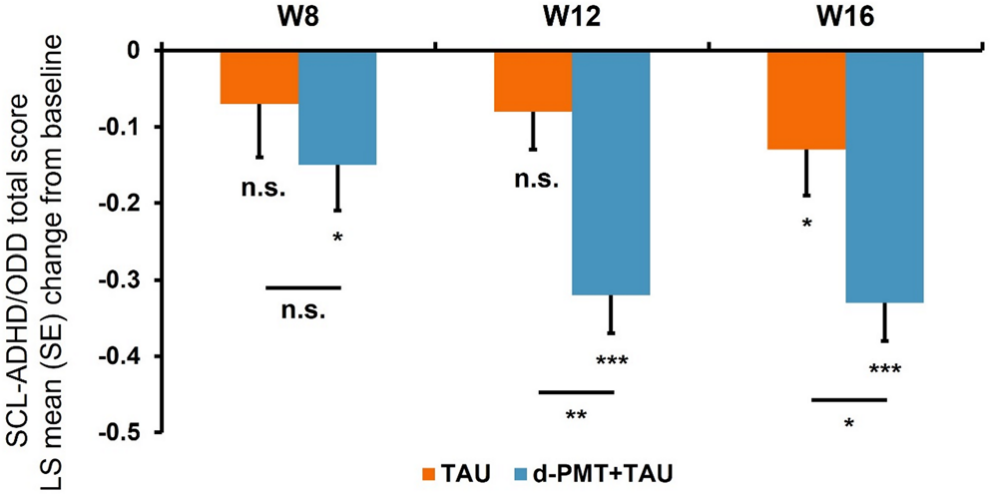
Time course of the change from baseline in the primary efficacy outcome variable. *p < 0.05; **p < 0.01; ***p < 0.001. d-PMT = digital parent management training; LS = least squares; n.s. = not significant; SCL-ADHD/ODD = Symptom Checklist-Attention-Deficit/Hyperactivity Disorder/Oppositional Defiant Disorder; SE = standard error; TAU = treatment as usual; W = week

The mean changes from baseline in the SCL-ADHD/ODD mean item score were greater in the dPMT + TAU group compared to the TAU group at all timepoints (Online Resource 7). The LS mean changes from baseline were statistically significant at all timepoints in the dPMT + TAU group but only at W16 in the TAU group (Fig. 2, Online Resource 8). The between-group comparisons of LS mean (95% CI) changes from baseline yielded statistically significant differences in favor of the d-PMT + TAU group at W12 (0.244 [0.095–0.393]; *p* = 0.0015) and W16 (0.199 [0.046–0.353]; *p* = 0.0115) (Fig. 2; Table 2). At both time points, the effect size was in the medium range (Table 2). Sensitivity analyses of the between-group comparisons under the not missing-at-random assumption yielded results consistent with those of the primary analysis.

**Table 2:** Between-group differences for the primary and secondary efficacy outcome variables

	Difference in LS means	95% CI	t-value	*p*-value (df = 117)	Cohen’s d
ADHD + ODD symptoms (SCL-ADHD/ODD)					
W8	0.076	-0.105–0.256	0.83	0.4084	0.148
W12	0.244	0.095–0.393	3.24	0.0015*	0.735
W16	0.199	0.046–0.353	2.57	0.0115*	0.483
**ADHD symptoms (SCL-ADHD)**					
W8	0.037	-0.143–0.217	0.40	0.6872	0.019
W12	0.222	0.063–0.380	2.76	0.0066*	0.594
W16	0.161	0.007–0.315	2.07	0.0409*	0.370
**ODD symptoms (SCL-ODD)**					
W8	0.171	-0.072–0.414	1.40	0.1654	0.329
W12	0.298	0.116–0.481	3.24	0.0015*	0.770
W16	0.291	0.078–0.505	2.70	0.0079*	0.549
**Impairment (SCL-ADHD part F)**					
W8	0.306	0.094–0.519	2.86	0.0050*	0.463
W12	0.432	0.222–0.641	4.07	< 0.0001*	0.835
W16	0.252	0.033–0.472	2.28	0.0244*	0.275
**Positive parenting (FPNE pos.)**					
W8	-0.104	-0.226–0.017	-1.70	0.0920	-0.497
W12	-0.163	-0.302– -	-2.33	0.0212*	-0.676
W16	-0.172	0.025 -0.298– -0.045	-2.69	0.0081*	-0.744
**Negative parenting (FPNE neg.)**					
W8	0.148	0.001–0.295	2.00	0.0478*	0.467
W12	0.185	0.038–0.332	2.49	0.0142*	0.626
W16	0.153	-0.007–0.314	1.89	0.0607	0.485
**Family strain (FSI)**					
W8	1.772	0.455–3.089	2.66	0.0088*	0.623
W12	1.722	0.300–3.145	2.40	0.0181*	0.649
W16	2.760	1.189–4.330	3.48	0.0007*	0.749

ADHD = attention-deficit/hyperactivity disorder; CI, confidence interval, df = degrees of freedom, FPNE neg. = negative-inadequate parenting scale from the Assessment Scale of Positive and Negative Parenting Behavior; FPNE pos. = positive parenting scale from the Assessment Scale of Positive and Negative Parenting Behavior; FSI = Family Strain Index; ODD = oppositional defiant disorder; LS = least squares, SCL-ADHD = Symptom Checklist-Attention-Deficit/Hyperactivity Disorder; SCL-ADHD/ODD = Symptom Checklist-Attention-Deficit/Hyperactivity Disorder/Oppositional Defiant Disorder; SCL-ODD = Symptom Checklist-Oppositional Defiant Disorder; W = week; * = statistically significant

**Fig. 3:**
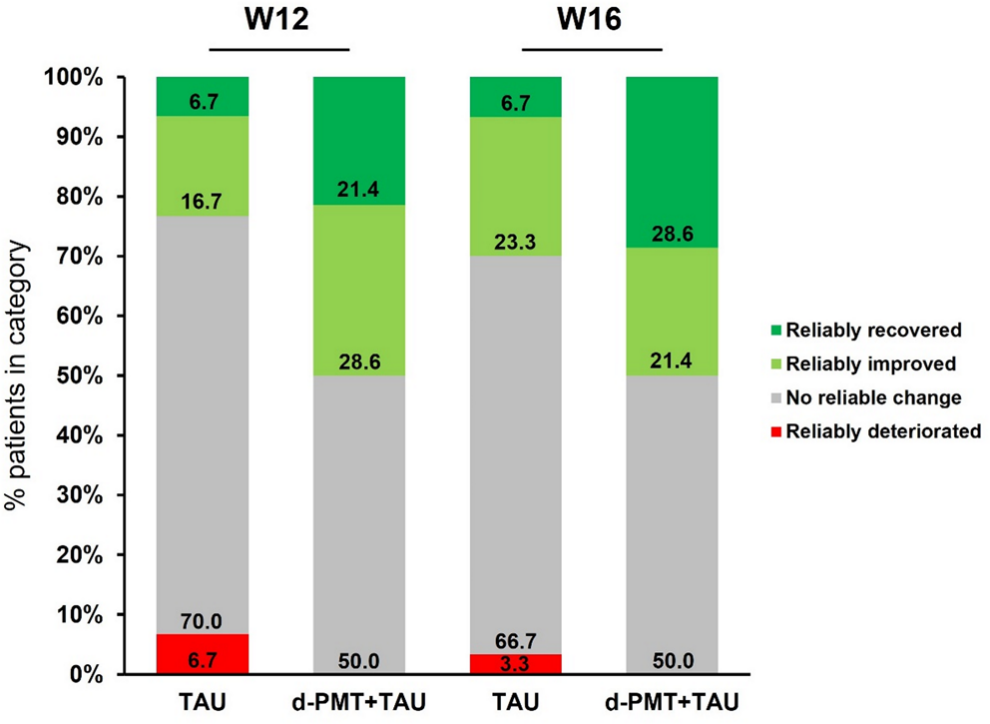
Clinical significance based on the reliable change index for the primary efficacy outcome variable. The analysis was restricted to children with an SCL-ADHD/ODD mean mean item score ≥ 1.0 at baseline. Children with missing values at W12 and/or W16 were classified as “no reliable change”. d-PMT = digital parent management training; SCL-ADHD/ODD = Symptom Checklist-Attention-Deficit/Hyperactivity Disorder/Oppositional Defiant Disorder; TAU = treatment as usual; W = week

Based on the RCI, the proportions of children who were classified as reliably recovered were numerically higher in the d-PMT + TAU group vs. the TAU group at W12 and W16 in both the ITT population (Online Resource 9, W16: 20.6% vs. 6.5%) and the population that only included children with an SCL-ADHD/ODD mean item score ≥ 1.0 at baseline (Fig. 3, d-PMT + TAU: *n* = 28, TAU: *n* = 30, W16: 28.6% vs. 6.7%). Overall, 50% of the patients in the d-PMT + TAU group were classified as reliably recovered or improved at W16. Of note, one child in the TAU group was classified as reliably deteriorated.

Response analyses showed that, in the ITT population, the proportion of children who either reliably improved or reliably recovered was numerically higher in the d-PMT + TAU group compared to the TAU group at both W12 (35.3% vs. 22.6%; *p* = 0.394) and W16 (35.3% vs. 25.8%; *p* = 0.576) (Online Resource 9). Of note, the d-PMT + TAU group included six children with an SCL-ADHD/ODD mean item score < 1.0 at baseline, whereas this was the case for only one child in the TAU group. Restricting the analysis to patients with an SCL-ADHD/ODD mean item score ≥ 1.0 at baseline yielded numerically larger between-group differences (W12: 50.0% vs. 23.3%, *p* = 0.066; W16: 50.0% vs. 30.0%, *p* = 0.198) (Fig. 3).

### Secondary efficacy outcomes

#### Parent rating scales

Mean and LS mean changes from baseline for the secondary efficacy outcomes ADHD symptoms (SCL-ADHD), ODD symptoms (SCL-ODD), functional impairment (SCL-ADHD part F), parenting behavior (FPNE) and family strain (FSI) are shown in Online Resources 7 and 8, respectively. Significant treatment effects in favor of dPMT + TAU compared to TAU were observed for all secondary outcomes, with mostly moderate to large effect sizes at W12 and W16 (Table 2).

#### Therapeutic measures taken during the study

Therapeutic measures taken during the study are summarized in Online Resource 10. The proportion of patients with at least one treatment change was higher in the TAU group compared to the d-PMT + TAU group (25.8% vs. 17.6%), as was as the number of newly started treatments (ten vs. five treatments). Occupational therapy was the most common newly started ADHD/ODD treatment during the study.

#### Clinical global impression

CGI-S/CGI-I data were available for 31 patients per group at the final study visit. Mean ± SD CGI-S scores were comparable between the two treatment groups (d-PMT + TAU: 4.0 ± 0.8; TAU: 4.1 ± 1.0). The mean ± SD CGI-I score at the final study visit was 3.1 ± 0.7 in the dPMT + TAU group and 4.2 ± 0.6 in the TAU group. In the d-PMT + TAU group, 23 children showed an improvement (much improved: *n* = 6 [17.6%]; minimally improved: *n* = 17 [50.0%]); in contrast, only 3 (9.7%) children in the TAU group showed minimal improvement, and no child showed much improvement. The proportion of patients with no change was numerically higher in the TAU group (*n* = 21 [67.7%]) compared to the d-PMT + TAU group (*n* = 8 [23.5%]). No patient in the d-PMT + TAU group showed a worsening of symptoms, whereas symptoms worsened in seven patients in the TAU group (minimally worse: *n* = 6 [19.4%]; much worse: *n* = 1 [3.2%]).

#### Child’s global impression at study end

At the final study visit, the child’s global impression was assessed for 31 patients. Of these, 14 patients (45.2%) noticed that the parents tried to do something different, whereas 17 patients (54.8%) did not notice a difference. Among the patients who noticed a change in the parents’ behavior, 10 patients (71.4%) had a good feeling about these experiences/impressions and four patients (28.6%) had neither a good nor a bad feeling.

### Safety outcomes

Overall, 10 AEs were reported for eight persons (12.3%). In the d-PMT + TAU group, four AEs occurred in three patients (8.8%) and two AEs occurred in two participants (5.9%). In the TAU group, four AEs occurred in three patients (9.7%). No AEs were reported for other family members. None of the AEs was assessed as related to the study treatment and none of the AEs were serious. No participant/patient prematurely terminated study participation due to an AE. The most frequent AEs were those belonging to the MedDRA system organ class ‘social circumstances’ (d-PMT + TAU: two AEs, TAU: four AEs). Such AEs resulted from changes at school (e.g., illness of a teacher, close friend transferred to another school) or in the family (e.g., job change of a parent, death of a relative).

In the d-PMT + TAU group, seven device deficiencies were reported by seven participants. These device deficiencies were subject to bug fixing implemented in subsequent app releases. None of these device deficiencies caused an AE or might have caused a serious AE.

## Discussion

This is the first randomized controlled trial to evaluate the use and efficacy of a newly developed mobile-based d-PMT in children aged 4 to 11 years with a clinical diagnosis of ADHD, referred for treatment of externalizing symptoms (ADHD symptoms with or without oppositional behavior), and without pharmacological and/or behavioral therapy. In contrast to many other PMTs, this d-PMT was tailored to the individual needs of the parents and the specific challenges within the family. Moreover, therapeutic homework assignments, and monitoring of the transfer of the interventions to the daily family routine as well as tailored feedback to the parent regarding successful transfer were incorporated to enhance parent engagement and the transfer to everyday family life.

The usage data with an average total usage time of more than 8 h (499 min) and the median training plan completion rate of 85% suggest a high level of engagement with the program, comparable to face-to-face PMTs [35] and better than other d-PMTs (e.g [11, 36–38].

For the primary outcome (parent-rated combined ADHD + ODD symptoms), statistically significant and moderate to strong treatment effects were found for d-PMT + TAU compared to TAU (effect size of 0.74 at W12 and 0.48 at W16). These effect sizes are comparable to, or exceed, those found for face-to-face PMTs (e.g [1, 2])., Direct comparisons of d-PMTs that incorporated therapist-supported interventions with face-to-face interventions also showed comparable efficacy for both treatment approaches [11–13]. In the d-PMT used in this study, additional expert support was available via the support function but was used less than five times. Thus, the results of this study show that significant effects are possible for self-directed interventions without substantial therapist support. Overall, 50% of the patients in the d-PMT + TAU group were classified as reliably recovered or improved at W16, demonstrating the clinical relevance of the achieved treatment response.

For both ADHD symptoms and oppositional behavior only, as well as functional impairment of the child, treatment effects were similar to those observed for the primary outcome. With respect to the parents, moderate treatment effects were observed for parenting behavior, as expected for PMTs, and for parental stress. The latter observation is particularly noteworthy given the known correlation between parental psychopathology and child symptomatology and the general reciprocity between the externalizing symptoms of affected children and their parents’ stress [39–41]. Thus, the observed treatment effects support the role of PMT as an intervention addressing the family as a system. Along these lines, positive effects were found on clinicians’ global impression ratings at the end of the study based on a joint feedback session with the participating parent and the child. Overall, the observed effects are in line with previous meta-analyses that mostly reported at least small effects on similar outcome parameters [6–9]. However, the trials included in these meta-analyses included patient samples with varying clinical levels of disruptive behavior and with different diagnoses. Overall, this study demonstrates the broad effects of the d-PMT on child symptoms and functioning, as well as on parenting behavior and family stress. Further analyses are planned to examine the mediating mechanisms of these outcomes.

Given the randomized controlled design and the low dropout rate over the course of 16 weeks, this study generally demonstrates a high methodological standard. However, the study also has some limitations. The main limitation is the lack of blinding of the parents’ ratings, as is the case in most PMT studies, which may result in an inflation of treatment effects due to parental bias [1]. However, treatment effects were also found for the clinical global impression rated by the (also unblinded) clinician, and roughly half of the children reported changes in their parents’ behavior after being asked in a non-suggestive fashion. Moreover, treatment effects of PMTs were found both in unblinded and possibly blinded measures [1]. Nevertheless, future studies should also integrate ratings from blinded clinicians or, ideally, compare two active treatments using ratings from parents blinded to the specific study hypotheses. A further limitation is the high proportion of parents with advanced educational qualifications, which may have contributed to the positive effects of the intervention. Further studies are needed to determine whether the results can be generalized to parents with lower levels of education. Another limitation is the lack of follow-up data to assess the stability of treatment effects. Follow-up data may also improve the validity of unblinded parent ratings, as stable unblinded parent ratings at follow-up may provide further evidence that the observed treatment effects at least reflect a sustained effect on the parental perception of the child’s behavior problems. A further limitation is the lack of analyses on moderators (e.g., sex, age and severity of symptoms) and mediators of treatment effects (e.g., change in parenting, app usage or successful therapeutic homework assignments). Further studies on larger samples are needed to address these questions. The results of this study are limited to patients with a clinical diagnosis of ADHD and without medication or intensive psychotherapy. Yet, this setting reflects current recommendations for PMTs as early intervention, particularly for preschool or school-aged children. The effects on children with ODD or with children at risk for ADHD or ODD will be analyzed in subsequent studies.

In conclusion, our findings indicate that this novel mobile application may be used to reduce the current gap in the overall coverage with PMTs as an effective and safe tool in the treatment of ADHD and other externalizing disorders. Thus, the d-PMT may improve the multimodal treatment of children with ADHD in routine clinical practice.

## Electronic supplementary material

Below is the link to the electronic supplementary material.

Supplementary Material 1

## Data Availability

Data are available upon reasonable request to the corresponding author.
