# Supplementary material for: Efficacy of a mobile-based self-directed parent management training for parents of children with attention-deficit/hyperactivity disorder with or without oppositional defiant disorder– a randomized controlled trial
Source: Eur Child Adolesc Psychiatry. 2025 Jun 27;34(12):3973–85. doi: 10.1007/s00787-025-02799-2 (PMC12743068; doi:10.1007/s00787-025-02799-2)
Supplement: Supplementary file 1 — Supplementary Material 1 [file 787_2025_2799_MOESM1_ESM.pdf]

Supplementary Material

European Child & Adolescent Psychiatry

Efficacy of a mobile-based self-directed parent management training for parents of children with attention-deficit/hyperactivity disorder with or without oppositional defiant disorder – a randomized controlled trial

Döpfner*, M.[1,2], Görtz-Dorten*, A[1,2], Häge, A.[3], Handermann, F.[3], Sulprizio, L.[1,2], Hellmich, M.[4,5], Vogel, D.[6], Ruhmann, M. [7], Althen, H.[6], Karus, M.[6], Banaschewski, T[3,8].

* shared first authorship

[1] Centre of Child and Adolescent Cognitive Behavior Therapy (CEKIP), Faculty of Medicine and University Hospital Cologne, University of Cologne, Cologne, Germany

[2] Department of Child and Adolescent Psychiatry, Psychosomatics and Psychotherapy, Faculty of Medicine and University Hospital Cologne, University of Cologne, Cologne, Germany

[3] Department of Child and Adolescent Psychiatry and Psychotherapy, Central Institute of Mental Health, Medical Faculty Mannheim, Heidelberg University, Mannheim, Germany

[4] Institute of Medical Statistics and Computational Biology, Faculty of Medicine and University Hospital Cologne, University of Cologne, Cologne, Germany

[5] University Medical Center Göttingen, Department of Medical Statistics, Göttingen, Germany

[6] Medigital GmbH, Iserlohn, Germany

[7] MEDICE Arzneimittel Pütter GmbH & Co. KG, Iserlohn, Germany

[8] German Center for Mental Health (DZPG), partner site Mannheim-Heidelberg-Ulm, Germany

Corresponding author

Manfred Döpfner, University of Cologne, Cologne, Germany

manfred.doepfner@uk-koeln.de

Online Resource 1

Clinical investigation plan amendments

The final Clinical Investigation Plan (CIP) (version 1.0, dated 22 March 2022) was amended several times during the study. Key changes to the CIP after trial commencement (first patient in) are summarized below.

Changes made in CIP version 4.0 (dated 03 March 2023)

- To facilitate further recruitment, an online setting was introduced, and the respective processes were described (recruitment by the treating physician/psychotherapist, eConsent, and study conduct by a central online center). In this context, telemedicine-based baseline and final visits at the onsite study sites were made possible to accommodate families with long travel distances.
- The validity of pre-existing SCL-ADHD/SCL-ODD questionnaires completed by the parents / participants, which could be used for the screening process, was extended from two weeks to eight weeks after consultation with the scientific leads.

Changes made in CIP version 5.0 (dated 20 November 2023)

- The exclusion criterion regarding severe parent-reported symptoms (scores > 2.0 on the SCL-ADHD [total scale] and/or on the SCL-ODD [part A]) at screening was removed. This cut-off was originally implemented to exclude children for whom primary pharmacotherapy is indicated. However, because pharmacotherapy was used as additional exclusion criterion, this upper cut-off appeared redundant and at the same time increased the risk of unnecessary screening failures.

Online Resource 2

Detailed description of the digital parent management training

General structure

The digital parent management training (d-PMT; brand name hiToco®) is a mobile application available for both Apple iOS and Android OS and can be downloaded from the Apple App Store or the Google Play Store. It is a CE-marked class I medical device software according to regulation (EU) 2017/745 on medical devices (MDR). During study conduct, the application was a CE-marked class I medical device according to Directive 93/42/EEC.

The d-PMT is a comprehensive, integrated, and personalized program consisting of five modules: (1) psychoeducation on characteristics, causes, and further course of ADHD/ODD, (2) psychoeducation on assessment and interventions, (3) coping with parental challenges (e.g., reducing own stress, improving own self-control, improving own organizational skills, handling partner problems), (4) strengthening family resources and the parent-child relationship, (5) solving child behavior problems at home (eFigures 1 and 2). Module 5 aims to improve ADHD/ODD symptoms in the family. Four typical problem situations are presented (homework problems, chaos in the child's room, disturbing others, temper tantrums) and the parent is asked to select the problem situation that most closely aligns with the main problem situation at home. The parent is then guided to analyze the problem, to understand coercive parent-child interaction and to set a general framework in this situation (stimulus management), to define behavioral rules for the child, to communicate the rules, to praise the child for adherence to the rules, to set negative consequences, and to use a token economy. To personalize the intervention, the parent is guided at the beginning to select the chapters from modules 1 to 4 according to their specific needs and to select the problem situation that most closely aligns with the main problem situation at home. Transfer tasks aim to help the parents to implement the interventions developed in modules 3 to 5 at home.

Depending on the feedback of the parents regarding implementation success, the parents were positively reinforced or received further support to overcome implementation barriers.

Several techniques are used to help parents acquire the specific skills described above (short articles, interactive ratings, video-based modeling, interactive exercises) (eFigure 2). The implementation of these skills in daily routine is supported by therapeutic homework assignments with reminders, monitoring of the implementation, identification and modification of barriers to implementation, and positive feedback for successful implementation.

The entries made in the different sections of the app are securely stored in the backend of the app and can be viewed, edited and deleted by the user at any time. In the following, the five modules of the d-PMT and its functions are explained in more detail.

Module 1 – Things to know about ADHD/ODD: This module takes a closer look at the characteristics and causes of ADHD/ODD as well as possible developmental processes. Classic problems, as well as strengths and positive characteristics, of children with ADHD/ODD are shown. Parents can individually indicate the characteristics and problem area of their child, which will be addressed in later articles. The spotlight model is then introduced to the parents.

Module 2 – Help with ADHD/ODD: In this module, parents are informed about diagnostics and treatment options for ADHD/ODD through articles and explanatory videos.

Module 3 – Mastering your own challenges: In this module, parents learn how to better structure everyday life and reduce their stress. Tools for creating weekly schedules (weekly planer) and analyzing stressful times and schedules help in this process. The routine plan helps to predefine strategies for recurring challenging situations with the child.

Module 4 – Activating strengths: This module focuses on taking a closer look at one's own strengths and weaknesses, those of the child and those of the family, and strengthening the positive relationship with the child. Parents work through this with the help of the spotlight model. In addition, a digital positive diary is introduced, which allows the documentation of positive observations and experiences with the child.

Module 5 – Solve behavioral issues: In this module, the child's behavioral problems are analyzed and worked on. To this end, the parents are first explained the vicious circle of parenting and shown how this can be overcome using a multi-stage problem-solving circle. Example videos show typical problem situations in the family and illustrate alternative response options. Additionally, the rule plan and points plan help parents transfer these concepts into everyday life. Furthermore, a digital behavioral diary is introduced, which is maintained throughout the intervention and can illustrate changes in behavior over time.

Registration

After downloading the app, users must enter a valid prescription code/access code to proceed with the registration process. Next, they enter a valid email address and choose a password. Finally, account creation is completed using two-factor authentication.

Creation of individual training plan

After the initial login, users are prompted to answer a total of 16 questions. In addition, users are asked to select one of four different problem areas. Based on these entries, an individual "training plan" is created, which sets the content and its sequence for app usage. This helps users navigate through the complex and extensive content step-by-step, allowing them to work through their training plan in a structured order while reducing the risk of being overwhelmed by an overload of information. Content that is not part of the training plan based on the user's initial entries will not be accessible until the user has completed the respective

module. While users are working through their training plan, the "Toco Chat" actively engages from time to time by asking questions to encourage users to reflect on exercises or their own behavior and experiences (therapeutic feedback loops), or simply by sending reminders.

Sections of the App

The d-PMT is divided into four sections: *Home*, *Knowledge*, *Tools*, and *Support* (eFigure 3).

Home: The home screen provides an overview of the content completed in the app by displaying the progress in the training plan as a percentage and showing module progress. Users can also easily click the "Continue Reading" button, which automatically takes them to the next article to be completed within the training plan.

Knowledge: The knowledge section contains the entire content of all five modules and is only accessible after the completion of the respective module within the training plan. The modules are divided into chapters, which are further divided into smaller articles. The articles include, for example, short texts or model videos of typical parent-child situations.

Tools: The Tools section serves as the active part for applying the learned knowledge in everyday life. The tools are not accessible from the beginning; instead, they are unlocked one-by-one after completion of specific modules or chapters in the training plan. When users open a tool for the first time, a brief tutorial is provided in a chat-like format. This ensures that parents fully understand the rationale behind each tool and how to use it in practice before they use it themselves. Users are offered eight tools that help apply the learned knowledge in everyday life:

- *My entries*: allows users to review and modify entries made throughout the training plan

- *Spotlight model*: helps users focus on everyday problems and look at them from different angles
- *Weekly schedule*: helps users structure the week, thereby reducing stress
- *Routine plan*: helps users define strategies to cope with challenging situations with their child
- *Positive diary*: promotes positive thoughts and behaviors towards the child and fosters positive and joyful interactions with the child
- *Rule plan*: helps users establish clear rules and appropriate responses to the child's behavior
- *Points plan*: reinforces newly learned behaviors in the child through a playful and child-friendly approach
- *Behavioral diary*: allows documentation of the child's behavior to identify behavioral patterns.

Support: The Support section provides users with access to customer support. Questions related to technical aspects or the content of the program can be submitted via email. Importantly, feedback is limited to the content of the app and does not include psychotherapeutic or other therapeutic advice.

Recommendations for use

In principle, there are no spatial or temporal requirements for the use of this program. However, it is recommended to use the d-PMT primarily in situations that allow engagement with the content and reflection without unnecessary interruption, for example at home in the evening. Parents are free to use the d-PMT at their discretion. However, regular use is recommended.

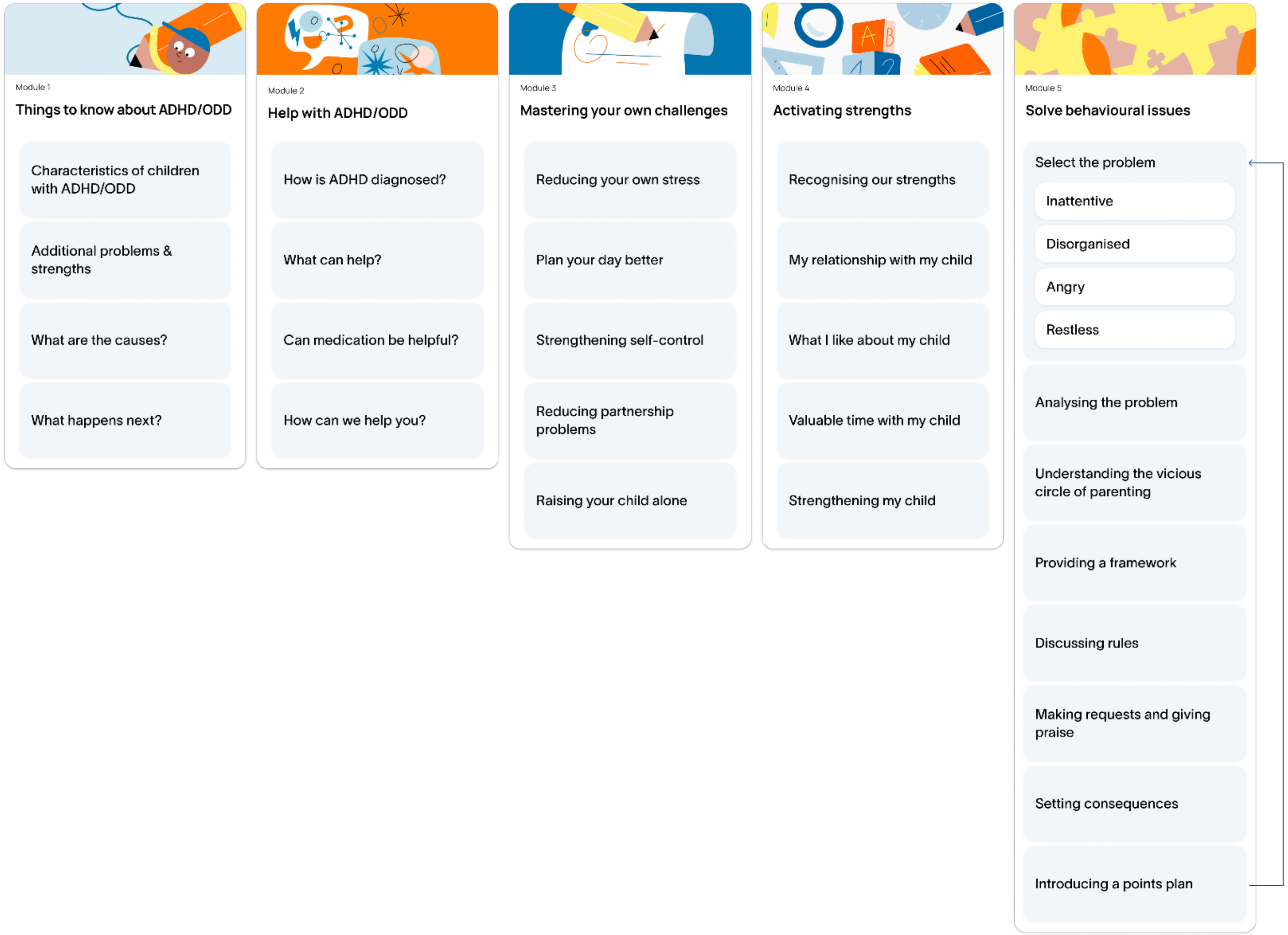

eFigure 1: Modules and chapters of the digital parent management training

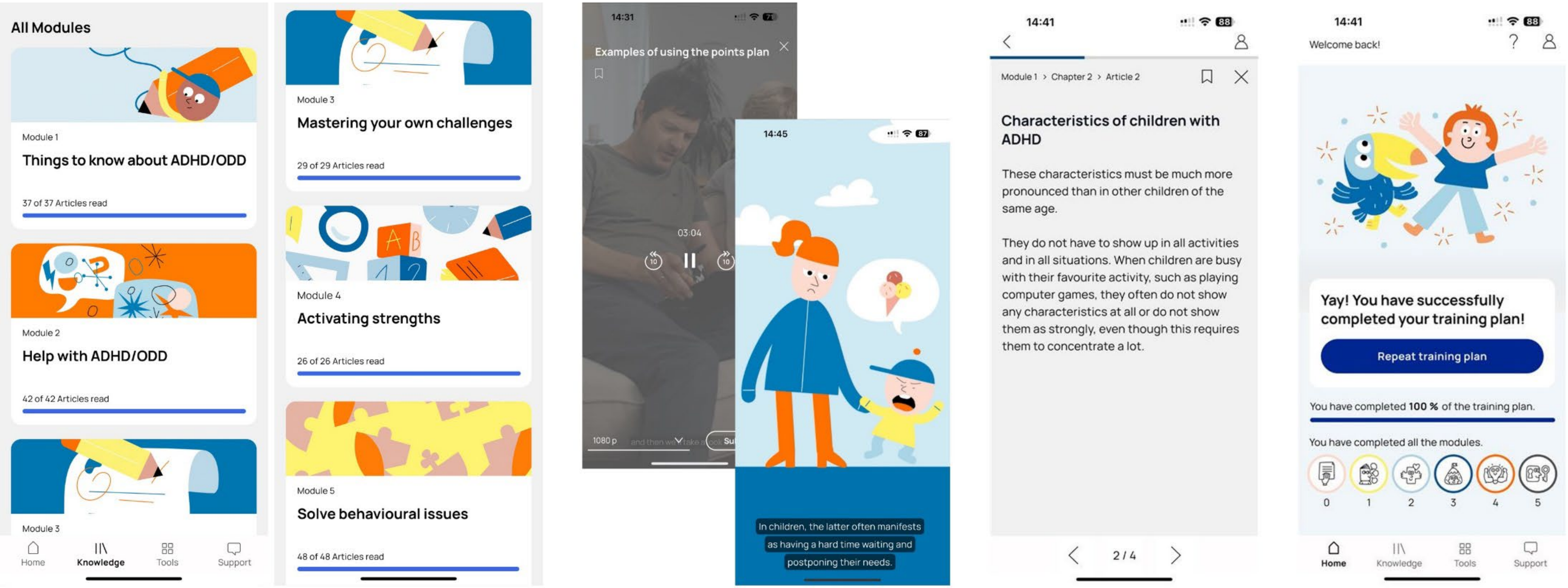

modules 1 - 5

model videos & cartoons

short articles

completed training plan

eFigure 2: Example screens from the English version of the digital parent management training showing the five modules and example contents

The content is provided in various formats, such as model videos and short articles, as the users work through their training plan.

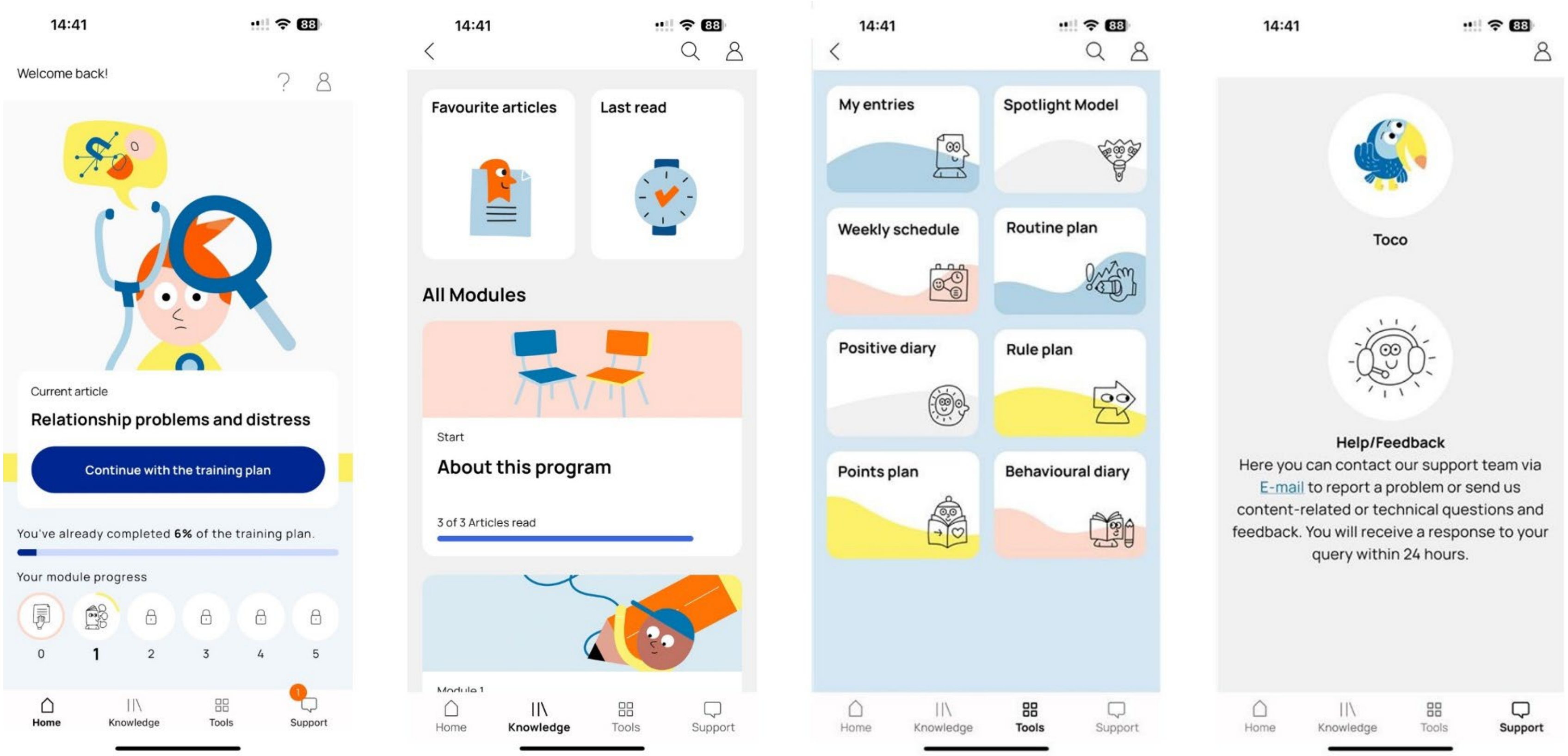

home knowledge tools support

eFigure 3: Example screens from the English version of the digital parent management training showing the four main sections: home, knowledge, tools, support

Online Resource 3

Description of efficacy outcome measures

SCL-ADHD/ODD scale

Both the SCL-ADHD and the SCL-ODD are based on the diagnostic criteria for ADHD and ODD, respectively, according to ICD-10 and DSM-5 and are part of the 3rd edition of the German Diagnostic System for Mental Disorders in Children and Adolescents (DISYPS-III) [1, 2]. Internal consistency of both rating scales is good to very good [1].

Factorial validity of the SCL-ADHD/ODD scale has been shown in several confirmatory factor analyses [2-4].

Internal consistency of the SCL-ADHD/ODD scale

The psychometric properties of the diagnostic system DISYPS-III were originally established for children and adolescents aged between 4 and 17 years based on a representative sample and a clinical sample [1]. For the SCL-ADHD and SCL-DB (disruptive behavior), n=713 and n=812 data sets, respectively, were available for the representative sample. For children with a clinical diagnosis of ADHD and/or ODD, n=1,294 and n=863 valid data sets were available for the SCL-ADHD and SCL-ODD, respectively, in the clinical sample. Because ADHD symptoms and oppositional-aggressive behavior often co-occur, the two clinical pictures are often grouped together as so called external or externalizing problem behavior [5]. As a result, hyperkinetic conduct disorder (F90.1) has been introduced into the ICD-10 classification. For children aged 4 to 11 years, the total ADHD scale of the SCL-ADHD (20 items) significantly correlates with the subscale for oppositional behavior of the SCL-DB (SCL-ODD, 8 items) in both the representative sample (r=0.67; n=321) and the clinical sample (r=0.39; n=1,138). This suggests that both scales can be combined. eTable 1 shows that the internal consistencies (Cronbach's α) of the combined scale SCL-ADHD/ODD (28 items) for the representative and

the clinical samples are very good (norm: α=0.95; clinic: α=0.93). The range of the item discrimination (r_{it}) for both samples is at least satisfactory, mainly good ($r_{it} \geq 0.33$). The means (±SD) of the SCL-ADHD/ODD mean item score for the representative and clinical samples are shown in eTable 2. In the representative sample, the average mean item score (±SD) is 0.59±0.51. In contrast, the average mean item score is notably larger in the clinical sample (1.46±0.58). Both values significantly differ from each other (p<0.001).

In the representative sample, SCL-ADHD/ODD mean item scores of 1.08 and 1.22 represented the 85th percentile and 90th percentile, respectively, for children aged 4 to 11 years. Scores above the 85th percentile may be considered indicative of clinically relevant symptoms. Children with a score at or above the 90th percentile can be considered in the clinical range. In the norm sample, these cut-off values correspond to the frequency with which children met the diagnostic criteria in the SCL-ADHD (parent-rated) [6]. Such cut-off values can be used to analyze whether changes in symptom severity are clinically relevant.

eTable 1: Internal consistency of the SCL-ADHD/ODD scale and range of the item discrimination (r_{it})

Sample	n	Number of items	Cronbach's α	r_{it}
Clinical	1,138	28	0.93	0.33 – 0.68
Representative	321	28	0.95	0.51 – 0.74

eTable 2: Mean and standard deviation for the SCL-ADHD/ODD mean item score in the norm and clinic sample

Sample	n	Mean	SD	t-value	p-value
Clinical	1,142	1.46	0.58	24.12	<0.001
Representative	322	0.59	0.51		

SD, standard deviation

Assessment Scale of Positive and Negative Parenting Behavior (FPNE)

The Assessment Scale of Positive and Negative Parenting Behavior (FPNE, German: Fragebogen zum positiven und negativen Erziehungsverhalten) contains 23 items divided into two scales: The positive parenting scale (FPNE pos.; 13 items), which assesses the frequency of positive, encouraging, and supporting parenting behavior, and the negative-inadequate parenting scale (FPNE neg.; 10 items), which assesses the frequency of negative, punishing, and inconsistent parenting behavior. Parents rated each item on a 4-point Likert scale (0 = never, 1 = rarely/sometimes, 2 = often, 3 = very often). Higher mean scores indicate more positive / negative parenting behavior. Internal consistency of both scales was assessed as satisfactory to good with a Cronbach's alpha of 0.78 for the FPNE neg. and 0.86 for the FPNE pos. [7].

Family Strain Index

The Family Strain Index (FSI) records the frequency of stressful family situations experienced by the parents within the last four weeks (parental stress). The FSI consists of six items, each of which is rated by the parent on a 5-point Likert scale (0 = never, 1 = hardly ever, 2 = sometimes, 3 = almost always, 4 = always). A higher sum score indicates larger perceived stress. Internal consistency of the FSI was found to be good with a Cronbach's alpha of .87 [8].

Online Resource 4

Throughout the entire study period, the proportion of missing participants remained below 10%.

eTable 3: Number of available participants for each assessment phase

	**TAU** N=31; n (%)	**d-PMT + TAU** N=34; n (%)	**Total sample** N=65; n (%)
Baseline visit	31 (100.0)	34 (100.0)	65 (100.0)
W0	31 (100.0)	34 (100.0)	65 (100.0)
W8	29 (93.5)	33 (97.1)	62 (95.4)
W12	28 (90.3)	32 (94.1)	60 (92.3)
W16	31 (100.0)	32 (94.1)	63 (96.9)
Final study visit	31 (100.0)	32 (94.1)	63 (96.9)

d-PMT, digital parent management program; TAU, treatment as usual

Online Resource 5

eTable 4: Extended baseline characteristics

	TAU (n=31)	d-PMT+TAU (n=34)	p-value[a]
**Child**			
At least one previous or current ADHD/ODD treatment, n (%)	14 (45.2)	18 (52.9)	0.705
Previous ADHD/ODD treatment[b], n			0.406
Total number of patients	7	4	
Child-focused intervention[c]	0	0	
Parent-focused intervention	0	0	
Drug treatment[d]	0	0	
Occupational therapy	4	3	
Neurofeedback	0	0	
Nutritional intervention	0	0	
Other	3	2	
Current ADHD/ODD treatment[b], n			0.296
Total number of patients	8	14	
Child-focused intervention[c]	1	2	
Parent-focused intervention	1	1	
Drug treatment[d]	0	0	
Occupational therapy	6	12	
Neurofeedback	0	0	
Nutritional intervention	1	0	
Other	3	1	
**Participating parent**			
Known ADHD diagnosis, n (%)	0 (0)	3 (8.8)	0.271
Number of further children in the household, mean±SD	1.0±0.8	1.1±0.8	0.782
Single parent, n (%)			0.410
Yes	1 (3.2)	4 (11.8)	
No	30 (96.8)	30 (88.2)	
Relationship status, n (%)			0.085
Stable relationship/married	29 (93.5)	31 (91.2)	
Single/unmarried	0 (0)	3 (8.8)	
Other	2 (6.5)	0 (0)	

ADHD = attention-deficit/hyperactivity disorder; d-PMT = digital parent management training; ODD = oppositional defiant disorder; SD = standard deviation; TAU = treatment as usual

[a] The p-values for differences in mean (e.g., age) are obtained by two-sided two-sample t-test. The p-values for differences in proportions (e.g., sex, diagnosis) are based on the chi-square-test for independence of the margins in a contingency table.

[b] Multiple meanings possible

[c] Child-focused interventions including cognitive behavioral therapy and other training

[d] Approved for ADHD treatment in Germany (i.e., methylphenidate, lisdexamfetamine, dexamphetamine, atomoxetine, guanfacine)

Online Resource 6

eTable 5: App usage throughout the study

	Period of use (days)	Logins/user (number)	Login duration (min)	Total usage (min)	Training plan completion rate (%)
n	21	21	21	21	29
Mean (SD)	119.4 (25.0)	35.0 (22.1)	14.3 (18.7)	498.6 (269.3)	78
Median	117.3	32	7.6	452.4	85
IQR	103.0 – 140.3	18.3 – 45.3	1.0 – 21.1	287.9 – 781.1	51 – 100
Range	67.1 – 157.1	5 – 95	0.2 – 130.4	104.0 – 939.5	5 – 100

IQR, interquartile range; SD, standard deviation

Online Resource 7

eTable 6: Change from baseline in primary and secondary efficacy outcome variables (raw values)

	TAU			d-PMT+TAU		
	**n**	**Mean (SD)**	**Median [IQR]**	**n**	**Mean (SD)**	**Median [IQR]**
**ADHD+ODD symptoms (SCL-ADHD/ODD)**						
W8	29	-0.083 (0.309)	-0.107 [-0.286; 0.107]	33	-0.132 (0.356)	-0.107 [-0.357; 0.071]
W12	28	-0.078 (0.299)	-0.036 [-0.321; 0.071]	32	-0.310 (0.330)	-0.286 [-0.393; -0.125]
W16	31	-0.144 (0.319)	-0.143 [-0.393; 0.107]	32	-0.314 (0.380)	-0.268 [-0.571; -0.018]
**ADHD symptoms (SCL-ADHD)**						
W8	29	-0.109 (0.309)	-0.150 [-0.250; 0.150]	33	-0.115 (0.382)	-0.050 [-0.300; 0.150]
W12	28	-0.127 (0.309)	-0.075 [-0.350; 0.050]	32	-0.327 (0.359)	-0.275 [-0.475; -0.100]
W16	31	-0.194 (0.293)	-0.150 [-0.350; 0.000]	32	-0.320 (0.384)	-0.200 [-0.600; -0.075]
**ODD symptoms (SCL-ODD)**						
W8	29	-0.017 (0.440)	0.000 [-0.250; 0.250]	33	-0.174 (0.508)	-0.125 [-0.375; 0.125]
W12	28	0.045 (0.413)	0.125 [-0.125; 0.250]	32	-0.270 (0.404)	-0.188 [-0.563; 0.000]
W16	31	-0.020 (0.510)	-0.125 [-0.500; 0.375]	32	-0.297 (0.498)	-0.250 [-0.500; 0.063]
**Impairment (SCL-ADHD part F)**						
W8	29	0.06 (0.39)	0.00 [0.00; 0.20]	33	-0.13 (0.43)	-0.20 [-0.40; 0.20]
W12	28	0.09 (0.43)	0.10 [-0.20; 0.40]	32	-0.29 (0.47)	-0.20 [-0.40; 0.00]
W16	31	-0.07 (0.52)	0.00 [-0.40; 0.40]	32	-0.21 (0.51)	-0.20 [-0.40; 0.20]
**Positive Parenting (FPNE pos.)**						
W8	29	-0.027 (0.214)	-0.077 [-0.154; 0.077]	33	0.105 (0.302)	0.000 [-0.077; 0.308]

	TAU			d-PMT+TAU		
	**n**	**Mean (SD)**	**Median [IQR]**	**n**	**Mean (SD)**	**Median [IQR]**
W12	28	-0.033 (0.246)	0.000 [-0.192; 0.115]	32	0.175 (0.353)	0.077 [-0.077; 0.385]
W16	31	-0.030 (0.279)	0.000 [-0.231; 0.154]	32	0.178 (0.279)	0.154 [-0.038; 0.385]
**Negative Parenting (FPNE neg.)**						
W8	29	-0.038 (0.285)	0.000 [-0.200; 0.200]	33	-0.188 (0.350)	-0.100 [-0.400; 0.100]
W12	28	-0.061 (0.290)	0.000 [-0.250; 0.100]	32	-0.259 (0.340)	-0.250 [-0.500; 0.000]
W16	31	-0.094 (0.282)	-0.100 [-0.300; 0.200]	32	-0.256 (0.380)	-0.350 [-0.500; 0.000]
**Family Strain (FSI)**						
W8	29	-1.4 (2.3)	-1.0 [-3.0; 0.0]	33	-3.1 (3.2)	-3.0 [-5.0; -1.0]
W12	28	-1.5 (3.1)	-1.0 [-3.5; 0.5]	32	-3.6 (3.2)	-4.0 [-5.0; -2.0]
W16	31	-1.6 (3.9)	-1.0 [-4.0; 0.0]	32	-4.3 (3.3)	-4.5 [-6.0; -2.5]

ADHD = attention-deficit/hyperactivity disorder; d-PMT = digital parent management training; FPNE neg. = negative-inadequate parenting scale from the Assessment Scale of Positive and Negative Parenting Behavior; FPNE pos. = positive parenting scale from the Assessment Scale of Positive and Negative Parenting Behavior; FSI = Family Strain Index; IQR, interquartile range; ODD = oppositional defiant disorder; SCL-ADHD = Symptom Checklist-Attention-Deficit/Hyperactivity Disorder; SCL-ADHD/ODD = Symptom Checklist-Attention-Deficit/Hyperactivity Disorder/Oppositional Defiant Disorder; SCL-ODD = Symptom Checklist-Oppositional Defiant Disorder; SD, standard deviation; TAU = treatment as usual; W8/12/16 = week 8/12/16

Online Resource 8

eTable 7: Within-group changes for primary and secondary efficacy outcome variables (LMMRM)

	TAU					d-PMT+TAU				
	LSM	95% CI	t-value	p-value	Cohen's d	LSM	95% CI	t-value	p-value	Cohen's d
**ADHD+ODD symptoms (SCL-ADHD/ODD)**										
W8	-0.073	-0.204 – 0.058	-1.11	0.2692	-0.267	-0.149	-0.272 – -0.026	-2.39	0.0184	-0.371
W12	-0.078	-0.186 – 0.030	-1.44	0.1537	-0.260	-0.322	-0.425 – -0.220	-6.24	<0.0001	-0.939
W16	-0.127	-0.236 – -0.017	-2.29	0.0236	-0.452	-0.326	-0.433 – -0.219	-6.01	<0.0001	-0.824
**ADHD symptoms (SCL-ADHD)**										
W8	-0.098	-0.228 – 0.033	-1.48	0.1416	-0.352	-0.134	-0.257 – -0.011	-2.16	0.0326	-0.301
W12	-0.119	-0.234 – -0.005	-2.06	0.0416	-0.411	-0.341	-0.450 – -0.232	-6.20	<0.0001	-0.911
W16	-0.174	-0.284 – -0.064	-3.14	0.0021	-0.660	-0.335	-0.443 – -0.227	-6.16	<0.0001	-0.834
**ODD symptoms (SCL-ODD)**										
W8	-0.013	-0.189 – 0.164	-0.14	0.8858	-0.039	-0.184	-0.351 – -0.017	-2.19	0.0308	-0.343
W12	0.024	-0.108 – 0.156	0.36	0.7211	0.108	-0.274	-0.400 – -0.149	-4.33	<0.0001	-0.667
W16	-0.010	-0.163 – 0.142	-0.14	0.8919	-0.040	-0.302	-0.451 – -0.152	-4.00	0.0001	-0.596
**Impairment (SCL-ADHD part F)**										
W8	0.121	-0.032 – 0.273	1.56	0.1209	0.143	-0.186	-0.330 – -0.042	-2.55	0.0119	-0.313
W12	0.101	-0.050 – 0.252	1.32	0.1889	0.214	-0.331	-0.474 – -0.188	-4.58	<0.0001	-0.607
W16	-0.004	-0.159 – 0.151	-0.05	0.9594	-0.137	-0.256	-0.408 – -0.105	-3.35	0.0011	-0.414
**Positive Parenting (FPNE pos.)**										
W8	-0.012	-0.010 – 0.076	-0.26	0.7952	-0.124	0.093	0.009 – 0.176	2.20	0.0295	0.347

	TAU					d-PMT+TAU				
	**LSM**	**95% CI**	**t-value**	**p-value**	**Cohen's d**	**LSM**	**95% CI**	**t-value**	**p-value**	**Cohen's d**
W12	-0.003	-0.103 – 0.097	-0.06	0.9505	-0.134	0.160	0.065 – 0.256	3.33	0.0012	0.496
W16	-0.009	-0.099 – 0.081	-0.19	0.8460	-0.107	0.163	0.075 – 0.251	3.67	0.0004	0.638
**Negative Parenting (FPNE neg.)**										
W8	-0.039	-0.145 – 0.068	-0.72	0.4717	-0.133	-0.187	-0.288 – -0.086	-3.66	0.0004	-0.537
W12	-0.069	-0.175 – 0.037	-1.29	0.2005	-0.210	-0.254	-0.356 – -0.152	-4.95	<0.0001	-0.764
W16	-0.098	-0.213 – 0.016	-1.70	0.0916	-0.332	-0.252	-0.364 – -0.140	-4.44	<0.0001	-0.674
**Family Strain (FSI)**										
W8	-1.354	-2.312 – -0.396	-2.80	0.0060	-0.598	-3.126	-4.030 – -2.222	-6.85	<0.0001	-0.988
W12	-1.904	-2.934 – -0.873	-3.66	0.0004	-0.498	-3.626	-4.606 – -2.645	-7.32	<0.0001	-1.109
W16	-1.567	-2.688 – -0.446	-2.77	0.0065	-0.427	-4.327	-5.427 – -3.226	-7.78	<0.0001	-1.324

ADHD = attention-deficit/hyperactivity disorder; CI, confidence interval; FPNE neg. = negative-inadequate parenting scale from the Assessment Scale of Positive and Negative Parenting Behavior; FPNE pos. = positive parenting scale from the Assessment Scale of Positive and Negative Parenting Behavior; FSI = Family Strain Index; ODD = oppositional defiant disorder; LMMRM = linear mixed model for repeated measures; LSM = least squares mean; SCL-ADHD = Symptom Checklist-Attention-Deficit/Hyperactivity Disorder; SCL-ADHD/ODD = Symptom Checklist-Attention-Deficit/Hyperactivity Disorder/Oppositional Defiant Disorder; SCL-ODD = Symptom Checklist-Oppositional Defiant Disorder; W8/12/16 = week 8/12/16

Online Resource 9

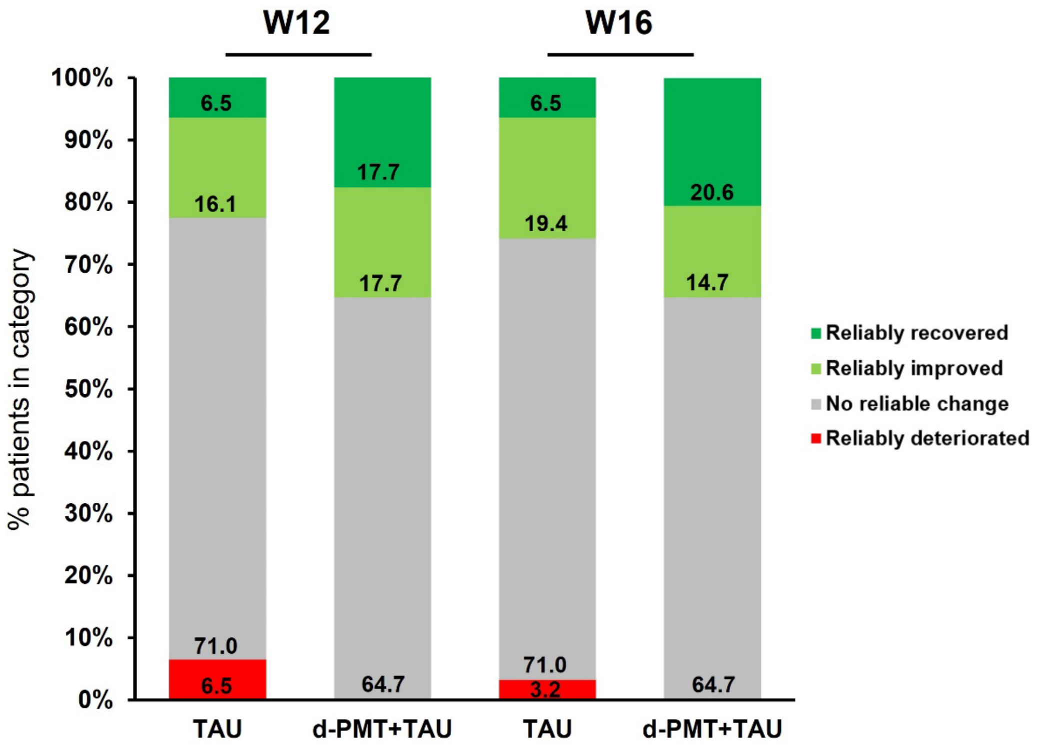

eFigure 4: Clinical significance based on the reliable change index for the primary outcome variable (ITT population)

Children with missing values at W12 and/or W16 were classified as "no reliable change".

d-PMT = digital parent management training; ITT = intention-to-treat; TAU = treatment as usual

Online Resource 10

eTable 8: Therapeutic measures taken during the study

	TAU (n=31)	d-PMT+TAU (n=34)
Patients with at least one treatment change, n (%)	8 (25.8)	6 (17.6)
Patients who started any ADHD/ODD treatment, n (%)	8 (25.8)	5 (14.7)
Patients who stopped any ADHD/ODD treatment, n (%)	1 (3.2)	2 (5.9)
Patients with at least one drug treatment change, n (%)	1 (3.2)	0 (0.0)
Newly started ADHD/ODD treatments on patient basis, n		
Total number of patients	8	5
Child-focused intervention[a]	2	3
Parent-focused intervention	1	0
Drug treatment[b]	2	1
Occupational therapy	4	0
Neurofeedback	0	0
Nutritional intervention	0	0
Other	0	1
Newly started ADHD/ODD treatments, n		
Total number of treatments	10	5
Child-focused intervention[a]	2	3
Parent-focused intervention	1	0
Drug treatment[b,c]	3	1
Occupational therapy	4	0
Neurofeedback	0	0
Nutritional intervention	0	0
Other	0	1
Drug treatments per active ingredient, n		
Methylphenidate	2	1
Lisdexamfetamine	1	0
Dexamphetamine	0	0
Atomoxetine	0	0
Guanfacine	0	0

[a] Child-focused interventions including cognitive behavioral therapy and other trainings
[b] Approved for ADHD treatment in Germany (i.e., methylphenidate, lisdexamfetamine, dexamphetamine, atomoxetine, guanfacine)
[c] Two patients initiated methylphenidate therapy, one of whom subsequently switched to lisdexamfetamine.

References

1. Döpfner M, Görtz-Dorten A (2017) Diagnostik-System für Psychische Störungen nach ICD-10 und DSM-5 für Kinder und Jugendliche – III [Diagnostic System for Mental Disorders in Childhood and Adolescence According to ICD-10 and DSM-5]. Hogrefe, Göttingen.
2. Thöne A-K, Junghänel M, Görtz-Dorten A, Breuer D, del Giudice T, Hanisch C et al (2022) Empirically based dimensions of externalizing symptoms in children and adolescents: a multitrait-multisource approach. J Psychopathol Behav Assess 44(3):844-861. https://doi.org/10.1007/s10862-022-09983-7
3. Rodenacker K, Hautmann C, Görtz-Dorten A, Döpfner M (2018) Evidence for the Trait-Impulsivity Etiological Model in a Clinical Sample: Bifactor Structure and Its Relation to Impairment and Environmental Risk. J Abnorm Child Psychol 46(4):659-669. https://doi.org/10.1007/s10802-017-0329-y
4. Thöne AK, Junghänel M, Görtz-Dorten A, Dose C, Hautmann C, Jendreizik LT et al (2021) Disentangling symptoms of externalizing disorders in children using multiple measures and informants. Psychol Assess 33(11):1065-1079. https://doi.org/10.1037/pas0001053
5. Steinhausen H-C, Döpfner M, Holtmann M, Philipsen A, Rothenberger A (2020) Handbuch ADHS: Grundlagen, Klinik, Therapie und Verlauf der Aufmerksamkeitsdefizit-Hyperaktivitätsstörung. Kohlhammer. https://doi.org/10.17433/978-3-17-034867-7
6. Döpfner M, Breuer D, Wille N, Erhart M, Ravens-Sieberer U (2008) How often do children meet ICD-10/DSM-IV criteria of attention deficit-/hyperactivity disorder and hyperkinetic disorder? Parent-based prevalence rates in a national sample--results of the BELLA study. Eur Child Adolesc Psychiatry 17 Suppl 1:59-70. https://doi.org/10.1007/s00787-008-1007-y
7. Holas V, Thone AK, Dose C, Gebauer S, Hautmann C, Gortz-Dorten A et al (2024) Psychometric properties of the parent-rated assessment scale of positive and negative parenting behavior (FPNE) in a German sample of school-aged children. Child Adolesc Psychiatry Ment Health 18(1):157. https://www.doi.org/10.1186/s13034-024-00850-9
8. Riley AW, Lyman LM, Spiel G, Döpfner M, Lorenzo MJ, Ralston SJ (2006) The Family Strain Index (FSI). Reliability, validity, and factor structure of a brief questionnaire for families of children with ADHD. Eur Child Adolesc Psychiatry 15 Suppl 1:I72-78. https://doi.org/10.1007/s00787-006-1010-0
